# Designing a Curriculum for the Disclosure of Medical Errors: A Requirement for a Positive Patient Safety Culture

**DOI:** 10.7759/cureus.6931

**Published:** 2020-02-10

**Authors:** Carolina Borz-Baba, Matthew Johnson, Vanitha Gopal

**Affiliations:** 1 Internal Medicine, Saint Mary's Hospital, Waterbury, USA; 2 Internal Medicine, Frank H. Netter MD School of Medicine Quinnipiac University, North Haven, USA; 3 Internal Medicine, McLeod Regional Health Center, Florence, USA

**Keywords:** disclosure of medical error, curriculum, patient safety

## Abstract

The Accreditation Council for Graduate Medical Education (ACGME) has endorsed the disclosure of adverse treatment events as a common program requirement for resident education and experience since July 2019. This article explores the residents' current attitudes and knowledge in the disclosure of medical errors and the efforts to design a more specific and effective educational program. We conducted a cross-sectional survey of medical residents toward this end. We observed that 62.5% of the residents were not familiar with the error-reporting process at our institution. General concerns about disclosing errors are related primarily to negative patient reactions (66.7%). The majority (58.3%) of the trainees' negative psychological experience after an unanticipated outcome resulting in harm has caused increased anxiety about future errors. To ensure a positive error-disclosure culture, the curriculum must include efforts to educate trainees on the error-reporting system and the disclosure process and should create an opportunity for the organization to establish programs and policies to guide practitioners through the process of disclosures.

## Introduction

Disclosure of medical errors is an important component of promoting a just medical culture. Patients want to be informed about the unanticipated outcomes that may occur during their care, and providers are required to become more comfortable with disclosing medical errors. Barriers to the disclosure of medical errors include unfamiliarity with the type of medical errors and their reporting process at an institution, inexperience with the disclosure process, and a negative organizational culture towards error transparency. Medical error disclosure training during residency advances physicians' knowledge, skills, and attitudes [1]. Organizational culture plays a fundamental role in promoting transparency towards the impact of medical errors on both patients and physicians. Patients who suffer adverse outcomes value the changes that organizations promote to avoid similar situations in the future. A disclosure-positive culture enhances the clinical application of knowledge acquired by the trainee [2]. Resident and organizational awareness of psychological distress experienced by healthcare practitioners following disclosure are critical in understanding the common emotional impact on physicians’ lives and can advance the necessity to establish organizational strategies to support all providers involved in disclosures [3].

## Materials and methods

Participants in this study were residents at the Yale Primary Care Residency Program, working in a community hospital in an underserved area. An anonymous paper-based survey was sent to 30 internal medicine trainees and four preliminary interns in February 2019. The objective of the survey was the evaluation of residents' knowledge and attitude toward medical errors. The survey also assessed residents' confidence in identifying the type of adverse events/errors and the barriers encountered in reporting them to the institution or in disclosure to patients. During the literature search, we identified several areas of essential value to structure our curriculum. Understanding the different categories of adverse events and recognizing the type of errors before reporting or disclosing is a basic requirement for practitioners [4]. This has to be complemented by the knowledge of error disclosure management plan to streamline the communication between patient, practitioner, and organization [5].

Formulating an effective disclosure message is paramount to foster patient-physician trust [5]. A successful message includes a genuine apology. However, this could be easily followed or preceded by compromising statements in which physicians would try to disown their mistakes by blaming others. Based on “the disclosure mistake to avoid”, we developed scripting that could be used by practitioners during communication with the patients [5]. Another domain investigated by our survey was the emotional impact of errors on physicians’ lives. We are aware that promoting a system that cares for the care provider is now a best practice recommendation [6]. The questions evaluated our residents' perception of common experiences following disclosures [3].

A 5-point Likert scale (very unlikely, unlikely, not sure, likely, and very likely) was used to assess residents' confidence in their knowledge of the types of adverse events that should be reported and their attitudes about the most common phrases used in disclosure messages.

## Results

Eighteen out of the total number of 34 residents (53%) completed the anonymous survey. The respondents included six postgraduate year-1 (PGY1) residents, three PGY2 residents, and nine PGY3 residents.

**Figure 1:**
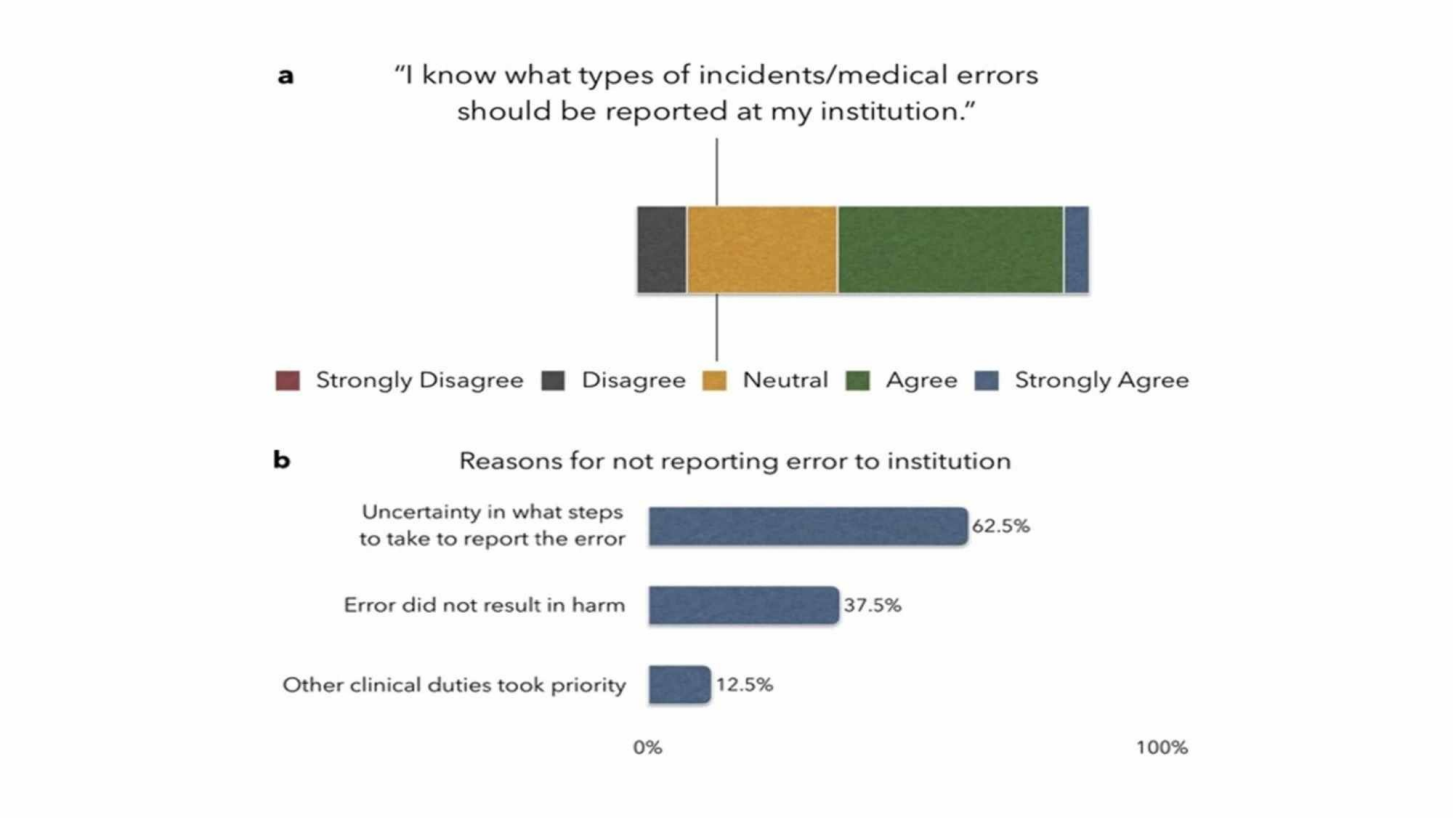
Residents' attitude toward reporting errors to the institution a: knowledge and level of confidence about reporting; b: reasons for not reporting

When asked if they felt confident about their knowledge of the types of incidents or errors that should be reported to the institution, 55.6% of residents answered "agree" or "strongly agree", 11.1% answered "disagree", and 33.3% responded "neutral". Uncertainty as to what steps need to be taken to report errors was the most commonly cited reason for not reporting errors (62.5%), followed by the error not resulting in harm (37.5%), and other clinical duties taking priority (12.5%) (Figure 1).

**Figure 2:**
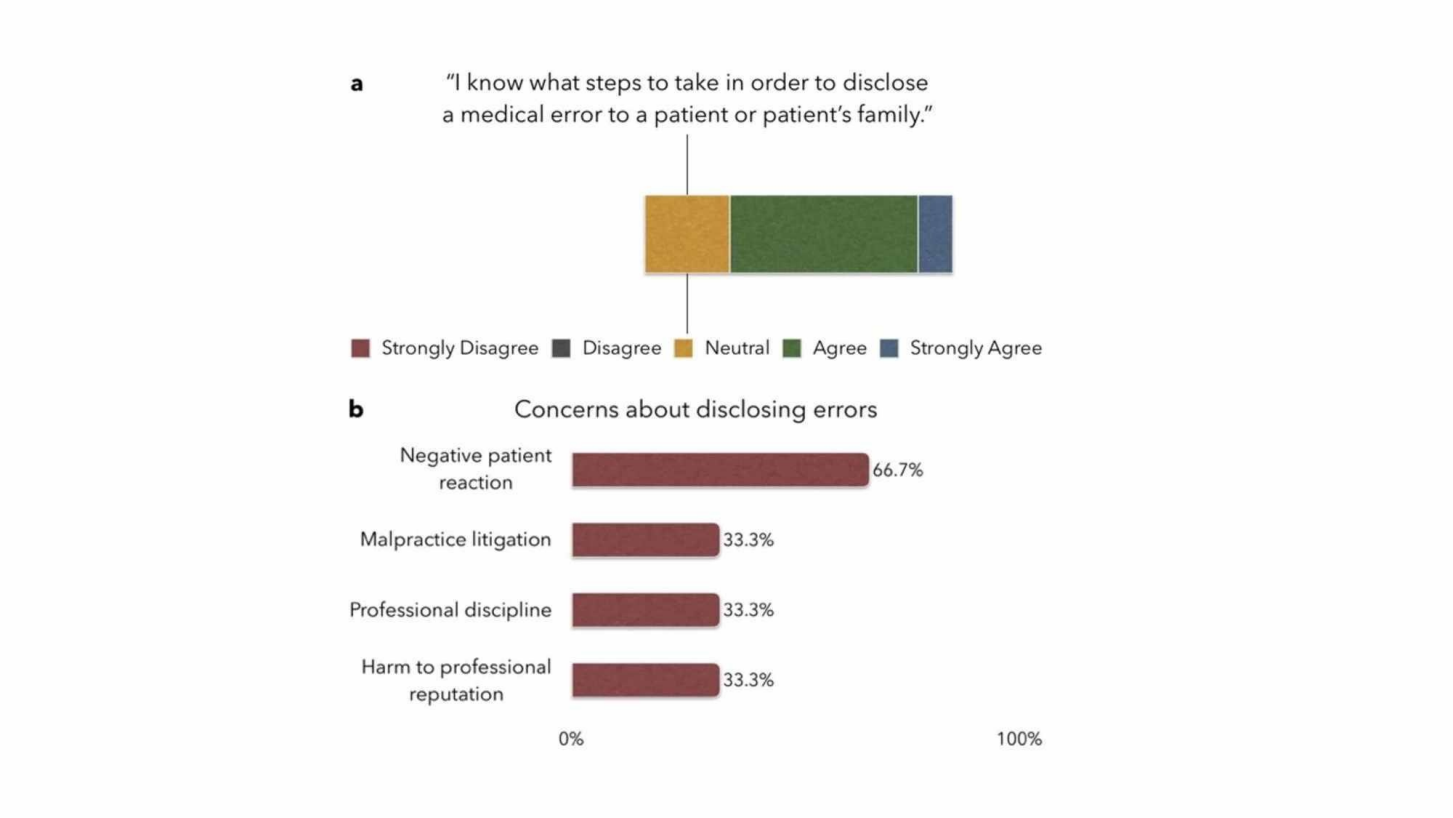
Residents' attitude toward disclosing errors to patients or patients' families a: knowledge and level of confidence about disclosing; b: concerns about disclosing

When asked if they felt confident about their knowledge of the steps to take in disclosing an error to a patient or patient's family, 72.2% of the residents answered "agree" or "strongly agree", and 27.8% responded "neutral", with none responding "disagree" or "strongly disagree". Residents' primary concern with disclosing errors to patients was a potential negative patient reaction (66.7%). Additional concerns about disclosing were malpractice litigation, professional discipline, and harm to professional reputation, with 33.3% of residents selecting each (Figure 2).

**Figure 3:**
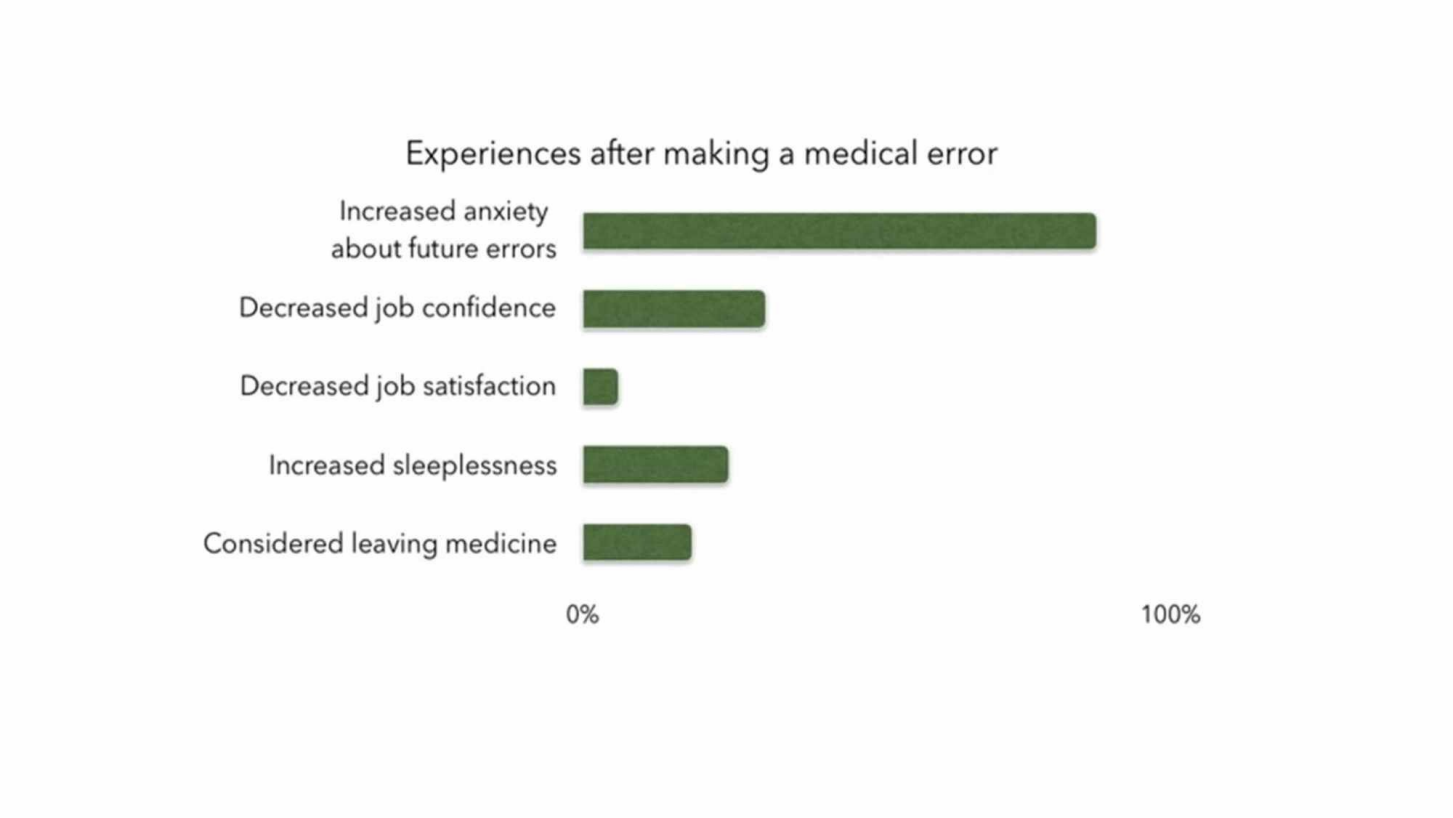
Residents’ experiences after making a medical error

An overwhelming majority (87.5%) of the residents reported increased anxiety after making a medical error, and 18.8% reported considering leaving medicine after making an error. Other experiences reported by residents after making medical errors included decreased job confidence (31.3%), increased sleeplessness (25.0%), and decreased job satisfaction (6.3%) (Figure 3).

**Figure 4:**
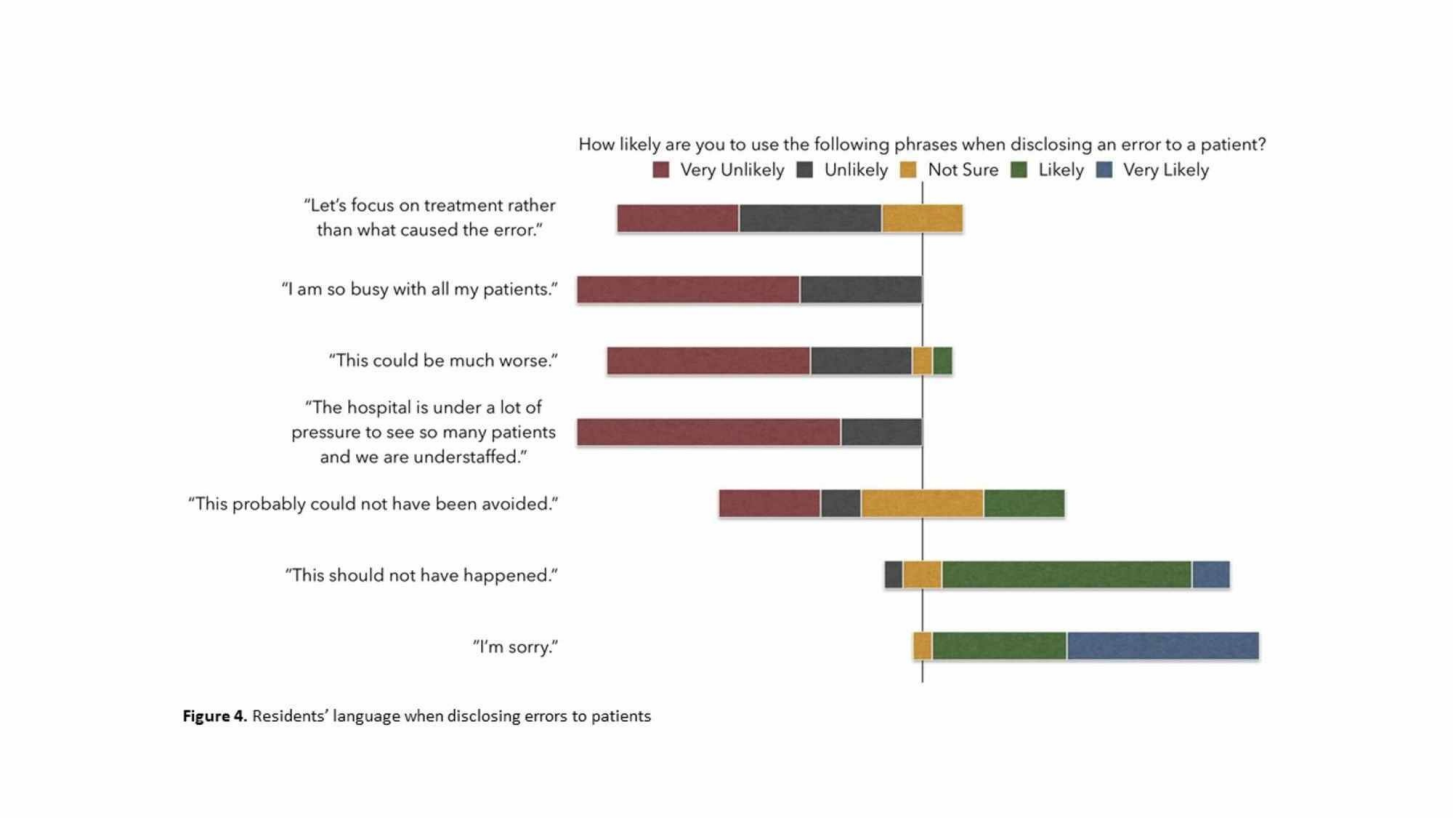
Residents' language when disclosing errors to patients

The majority of the residents reported being unlikely or very unlikely to use phrases such as "Let's focus on treatment rather than what caused the error" (76.5%) or "This could be much worse" (88.2%) when disclosing errors to patients or patients' families, and a large majority of residents responded that they were likely or very likely to use the phrases "This should not have happened" (83.3%) or "I'm sorry" (94.5%) (Figure 4).

## Discussion

Designing a curriculum on medical error disclosure is an opportunity to review the obstacles that residents encounter in reporting and disclosing errors, to create a more customized program, and to evaluate residents' performance in different areas of core competencies.

Educating residents on the types of adverse events and medical errors is the first step in preparing residents to be more proactive in using the institution’s reporting system. At our organization, adverse events are defined as "the failure of a planned action to be completed as intended or the use of a wrong plan to achieve an aim." This broad definition of unanticipated outcomes or adverse events, which encompasses medical errors, is easily acknowledged by residents and other practitioners, as demonstrated by our study. We believe that it is practical to maintain an inclusive taxonomy of "adverse events", which is less confusing to the trainees, with the complementary specific education on different types of adverse events and medical errors, which should be included in the curriculum [7].

Physicians report only 1% of adverse events and are less likely to report errors that did not result in harm to the patient, as seen in our study [8]. Lack of reporting near misses or "close calls" remains a failed opportunity to improve the system and prevent future harm. Enhanced education on the learning value of near misses and positive role-modeling among faculty is a fundamental part of advancing towards a high-reliability organization.

The organization's system of reporting events has to be well understood in order to be used effectively. With this survey, we took the opportunity to inquire about the physicians’ understanding of the error-reporting process. The institution's official reporting system has been actively promoted to healthcare practitioners since 2013. Our survey showed that residents were not familiar with the options available to report errors, which include in-person reporting during the patient safety meetings, direct communication with a quality representative, or anonymous reporting via Infonet. These results prompted us to include a formal education on the steps required to report adverse events and errors at our hospital in our curriculum recommendation.

Significantly, 72% of the residents were hypothetically familiar with the steps necessary to disclose medical errors. However, none of the physicians had undergone training in disclosure. There is a gap between the hypothetical attitude and real practice. It is unlikely that residents knew the components recommended by safe practices for disclosing unanticipated outcomes in depth [6]. Our hospital has a policy for disclosure of the outcome of care, which was reviewed in 2011, but there is no formal process in place that clarifies what information the patient communication should contain. This reveals the need for a more comprehensive program that addresses the pre-disclosure action plan, the content of the error disclosure, and the techniques to be adopted for delivering a well-formulated message. The concerns expressed by practitioners after disclosing errors are numerous, including negative patient reactions, harm to professional reputation, and fear of litigation. Our study revealed that trainees were primarily worried about the emotional impact that their message had on patients. Harm to professional reputation, fear of professional disciplines, and fear of litigation were equally distributed in our study and did not mirror the results from other reports where the main concern was fear of litigation [9].

Physician disclosures of a medical error could involve a particularly difficult conversation. Adequate training and pre-disclosure support prepare the physician to deliver a well-formulated message and acknowledge that a potential negative patient reaction is part of the disclosure conversation. Fear of litigation can highly affect the physician's attitude towards disclosure [4]. Trainees would benefit from being familiar with the variety of programs available throughout the country that credibly endorse the idea that disclosures are related to a decreased liability rate.

Increased anxiety about future errors is the most prominent feature that characterizes trainee’s negative psychological experience, followed by decreased confidence and increased sleeplessness, which is consistent with other reports in the literature [3]. A question that remained unexplored in many studies we reviewed was if physicians contemplated leaving medicine following unintended harm to the patient.

Almost 18% of our residents would consider changing their careers, demonstrating the potentially serious impact of one mistake on an inexperienced trainee. Providing care to the caregivers requires a specific organization system and policy to ensure that practitioners receive timely care, which includes compassion, respect, reassurance, and if required, medical care [6]. Our hospital is in the process of creating a support system for all practitioners who are involved in the consequences of unintentional harm.

Our study has several limitations. The survey was anonymous but did not achieve a 100% response rate. The study was a cross-sectional survey, which may have allowed the variability in training levels to influence the results. The results are not generalizable, but we believe they could still help in building a more specific curriculum. To encompass improvement in knowledge and attitude, we recommend the implementation of both lecture-based educational strategies and simulated patient-training sessions.

The lecture-based educational sessions would include an e-learning session that will review the definition and types of errors, the disclosure conversation process, and disclosure content. This would be mandatory for all medical residents in the first year of training. Yearly conferences will revisit the reporting system used at our institution and will involve a practice session with core teaching to promote a positive role-modeling approach. This conference will also present and discuss the pre-disclosure and post-disclosure support system developed with the participation of risk management. The standardized patient-simulation session will briefly review the content of the disclosure discussion and the message errors to avoid. This will be followed by the actual practice, reflection on the discussion, and feedback sessions on the performance. Simulation of real-life experience would allow trainees to become more confident with the conversation flow and prepare them to embrace an attitude or style that emphasizes preserving a trustworthy patient-doctor rapport.

To ensure continuous improvement of our curriculum, the residents would perform an evaluation of the effectiveness of the educational sessions at the end of each session.

## Conclusions

Developing a curriculum for the disclosure of medical errors can lay a solid foundation for assessing and improving residents’ clinical knowledge, system-based practice, and interpersonal and communication skills. It can also address both general and specific trainee concerns related to this issue, facilitate direct collaboration with the organization to develop support programs for practitioners involved in disclosures, and promote a positive patient safety culture.
